# Intestinal mucin-type *O*-glycans: the major players in the host-bacteria-rotavirus interactions

**DOI:** 10.1080/19490976.2023.2197833

**Published:** 2023-04-05

**Authors:** S.A. Raev, J.O. Amimo, L.J. Saif, A.N. Vlasova

**Affiliations:** a Center for Food Animal Health, Department of Animal Sciences, College of Food, Agricultural and Environmental Sciences, The Ohio State University, Wooster, OH, USA; b Department of Animal Production, Faculty of Veterinary Medicine, University of Nairobi, Nairobi, Kenya

**Keywords:** Rotavirus, mucus, mucins, histo-blood group antigens, sialic acid, sialyltransferases, decoy epitopes, attachment, binding

## Abstract

Rotavirus (RV) causes severe diarrhea in young children and animals worldwide. Several glycans terminating in sialic acids (SAs) and histo-blood group antigens (HBGAs) on intestinal epithelial cell (IEC) surface have been recognized to act as attachment sites for RV. IECs are protected by the double layer of mucus of which *O*-glycans (including HBGAs and SAs) are a major organic component. Luminal mucins, as well as bacterial glycans, can act as decoy molecules removing RV particles from the gut. The composition of the intestinal mucus is regulated by complex *O*-glycan-specific interactions among the gut microbiota, RV and the host. In this review, we highlight *O*-glycan-mediated interactions within the intestinal lumen prior to RV attachment to IECs. A better understanding of the role of mucus is essential for the development of alternative therapeutic tools including the use of pre- and probiotics to control RV infection.

## GRAPHICAL ABSTRACT

**Figure:**
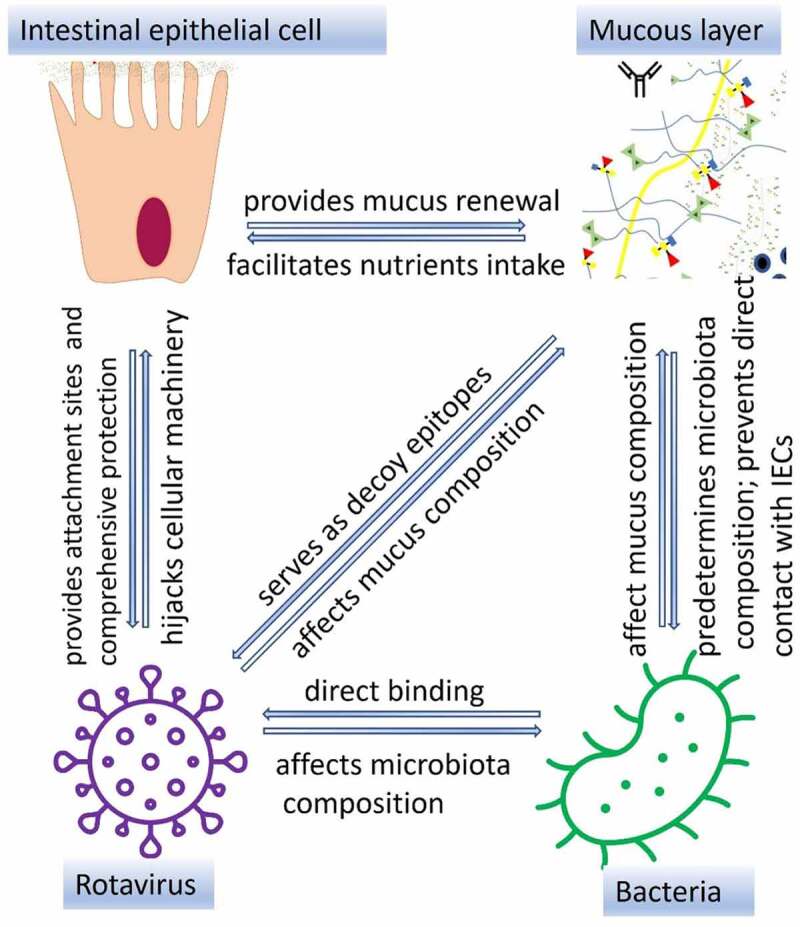

## Introduction

Rotaviruses (RVs) are the major causative agents of acute diarrhea in children and young animals globally.^1^ RV infects small intestinal epithelial cell (IEC) leading to villus atrophy, increased epithelial cell turnover, enhanced apoptosis, and formation of large vacuoles in enterocytes.^2^ To infect/enter IECs, RV binds several surface molecules such as sialic acids (SAs)^3,4^ and histo-blood-group antigens (HBGAs) in genotype-specific manner.^5,6^ However, presence of RV in the luminal content does not immediately result in its attachment on IECs, since they are protected from external environment by the mucus layer whose major organic component is represented by a variety of mucins, highly glycosylated molecules with complex oligosaccharides, *O*-glycans, including forementioned HBGAs with or without SA residue.^7^ Secreted mucins (not attached to IECs) are known to bind RVs thus serving as decoy receptors.^8^

The mucus layer is also a niche for the gut microbiota whose composition is predetermined by the host factors including the *O*-glycan profile.^9^ Growing evidence indicates that microorganisms including beneficial bacteria possess a wide range of factors enabling direct interactions with different components of the host mucus.^10^ There are several types of interactions between bacteria and mucus in the gut, including selective attachment, mucus degradation, and bacterial regulation of mucus production and composition.^8^ These bacteria-mucus interactions in the gut also influence the RV pathogenesis and disease outcome. The initial bacterial attachment to *O*-glycans present in mucus is provided by non-enzymatic glycan-binding proteins referred to as lectins.^11^ While presence of specific enzymes allows members of the gut microbiota to gradually degrade mucin *O*-glycans^12^, some bacteria stimulate *O*-glycan production by IECs.^13^ In addition, a wide range of bacteria have also been shown to produce glycans.^14^ Prior and our recent studies have demonstrated the presence of glycans recognized by human HBGA-specific monoclonal antibodies (Abs) on nonpathogenic phylogenetically diverse Gram-negative and Gram-positive bacteria.^15,16^ More importantly, bacteria expressing glycans have been shown to bind enteric viruses such as polioviruses^17^, noroviruses^15^ and RVs^16^ *in vitro* and *in vivo*. Thus, these bacterial glycans provide additional attachment sites for RV binding. Taken together, complex interactions within the intestinal mucus result in changes in composition/concentration of decoy receptors for RV thus affecting RV attachment and entry into IECs. This review focuses on the role of *O*-glycans in the host-microbiota-RV interactions within the GI tract and the implications of these findings on RV disease control strategies.

## O-glycans – are major organic constituents of the intestinal mucus

Mucus represents an ancient constituent of the epithelial barrier regulating crucial functions in a wide range of invertebrate and vertebrate species.^18^ In mammals, mucus is produced by specialized (goblet) epithelial cells scattered in the lining of the gastrointestinal (GI), the respiratory, and the reproductive tracts, as well as the ocular surface.^19^ Mucus is a complex of proteins, lipids, water, epithelial cells, leukocytes, mucins, and inorganic salts that form a gel-like structure.^20^ The mucus layer facilitates transport of nutritional components toward the epithelium in the GI tract and the exchange of gases within the respiratory tract. It maintains the viscoelastic properties of the reproductive tract and the preocular tear film. The mucus functions in the gut are mostly determined by mucins, glycoproteins encoded by the family of MUC genes.^21^

As part of host defense system, mucus within the GI tract serves as a physical barrier that reduces damage to IECs caused by food antigens, commensal microorganisms and the digestive secretions in the gut.^20^ It also protects IECs from being directly accessed and harmed by various pathogens including parasites, viruses and bacteria.^22^ Mucus antipathogenic function is achieved in part by the expulsion of pathogen-containing mucus controlled by peristaltic movements^23^ and contracting/swaying villi motions^24^ contributing to elimination of pathogenic organisms and other particles. Besides these “restrictive” functions, the gut mucus provides attachment sites for certain commensal bacteria promoting GI tract colonization.^25^ Thus, GI mucus is the primary site where interactions occur between the host and gut microorganisms.

Mucus comprises two layers whose composition and thickness vary throughout the intestine.^20^ It is the thinnest in the small intestine, while, in the large intestine the mucus layer is up to four times thicker.^20^ The inner layer is dense and non-penetrable to bacteria under normal physiological conditions. The outer layer, at least two times thicker, is loosely attached, allowing for bacterial binding.^26^ The permeability of the mucus layer has been found to be age-dependent in swine: it is more penetrable in 2-week old piglets compared to adult pigs.^27^ Besides, mucus density varies across the intestine,^28^ which correlates with regional bacterial abundance.^20^ Taken together, these unique properties of the mucus layers facilitate the corresponding functions of the small and large intestine: nutrient and water absorption (small intestine) and mostly water absorption (large intestine) and protection of IECs against microbial (pathogenic and nonpathogenic) invasion. The latter function is further provided by another important feature of the mucus layer, that of clearing the trapped material by luminal content movement with the average turnover time of the human small intestinal mucus gel and glycocalyx of 6–12 hours.^19^

**Figure 1.**
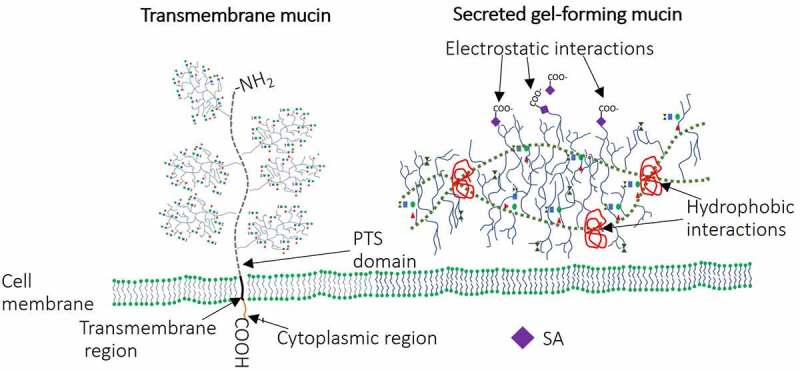
Schematic representation of secreted and transmembrane mucins. Transmembrane mucins are large and attached to IEC surface via transmembrane and cytoplasmic regions. Cysteine molecules widely present in PTS domains of secreted mucins form intra-disulfide bonds (hydrophobic interactions). In addition, the gel-like structure of secreted mucins is provided by electrostatic interactions within highly glycosylated regions.

Up to 95% of mucus consists of water, while the remaining 5% are the dry matter that contains cell debris, lipids, glycans and various proteins. The major organic part (80% of the total dry matter) of mucus consists of molecules of the mucin family, highly glycosylated proteins. The vast majority of mucin-type glycans are *O*-linked glycans; however, some N-linked glycans can be found on regions flanking the central protein backbone.^29^ All mucins are encoded by 22 genes and consist of 80% carbohydrates and 20% proteins.^30^ There are two structurally and functionally distinct groups of mucins: secreted (gel-forming and non-gel-forming) and membrane-associated. Secreted polymeric gel-forming mucins (MUC2, 5AC, 5B, 6 and 19) that create gel-like structure covering IECs are produced by goblet cells (Figure 1) and secreted monomeric non-gel forming mucins (MUC7 and MUC20) which are water soluble mostly found in bodily fluids such as saliva and tears where they lubricate and protect the eyes and mouth surfaces.^31,32^ Studies have shown that these structures have region-specific distribution. For example, MUC2 is more abundant in small intestine and colon while MUC5AC and MUC6 predominate the stomach and duodenum.^33–35^ Secreted mucins (gel forming) lubricate the intestinal mucosa, provide attachment sites for the commensal bacteria, regulate the gut microbiota composition and protect the IECs against pathogen invasion.^36^ Secreted mucin monomers aggregate via electrostatic and hydrophobic interactions resulting in a net-like polymeric gel structure (Figure 1) that facilitates all the mucus functions.^37,38^ Both, α-2,3 and α-2,6 N-acetylneuraminic acids (the major SAs) provide a critical role in gel-formation via electrostatic interactions (Figure 1).^37^ Mucin core hydrophobic domains further provide gel polymerization (Figure 1).^38^ The membrane-associated (transmembrane) mucins (MUC1, 3A, 3B, 4, 12, 13, 15, 16, 17, 18, 20, 21) do not form multimers but bind to IECs via short cytoplasmic domains and form glycocalyx (Figures 1 and 3).^39^ These mucins play a critical role providing connection between IECs and habitants of the outer mucus layer.^40^

**Figure 2.**
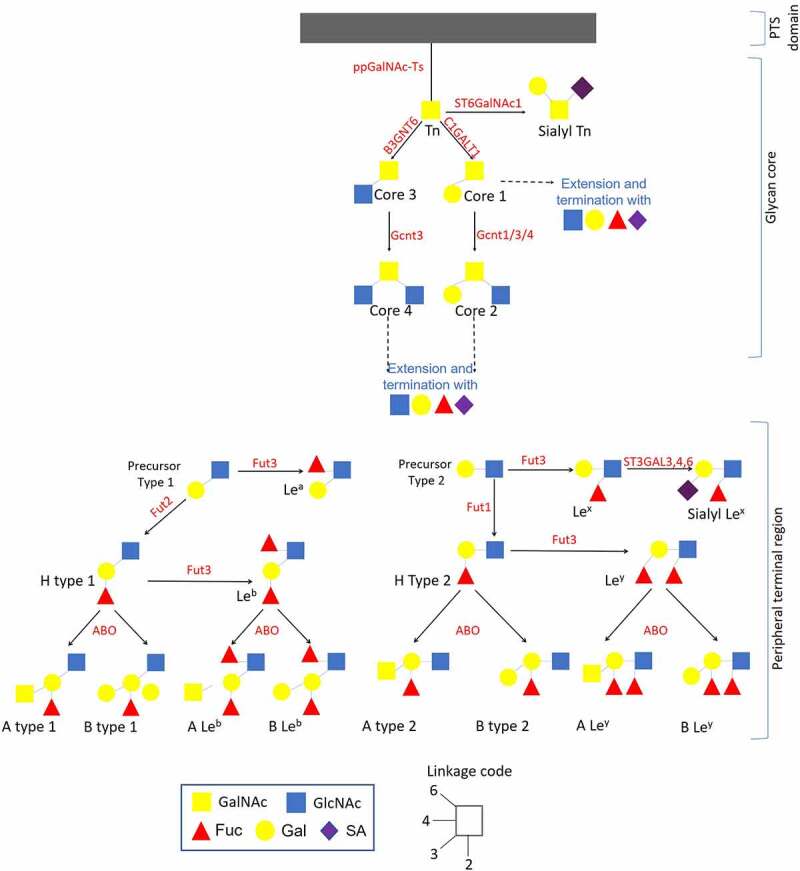
Schematic representation of the mucin family and their structure. Tandem repeat domains – enriched in proline (Pro), threonine (Thr) and/or serine (Ser) (PTS domain) are highly glycosylated [including N-acetylgalactosamine (GalNac), N-acetylglucosamine, fucose, galactose and sialic acid (SA)]. These *O*-linked glycan domains, represent 50% (w/w) of all mucins.^41^ Mucin glycosylation occurs within the endoplasmic reticulum and the Golgi apparatus; once glycosylated they are secreted on the apical surface of goblet cells.^42^ The initial step of mucin-type *O*-glycosylation in mammals (addition of GalNAc to PTS domain – Tn antigen) is provided by the activity of *N*-acetylgalactosaminyltransferases (ppGalnac-Ts) - enzymes which are encoded by one of 20 genes. This antigen is further extended by addition of GalNAc, galactose, N-acetylglucosamine and SA (provided by activity of different glycosyltransferases) leading to formation of four different glycan core types that can be included in the structure of MUC1, MUC2, MUC5AC, MUC5B, MUC6–8, MUC 11–13 and MUC 16.^39^ Activity of FUT2 (active only in secretor individuals) regulates the production of H-type 1 antigen while FUT1 enzyme is responsible for production of H-type 2 antigen (cell-associated antigen). The peripheral terminal region may be presented by l-fucose (Fuc), d-galactose (Gal), N-acetylgalactosamine (GalNac), N-acetylglucosamine (GlcNac) and SA residues, included in the structure of all HBGAs such as A, B, H, Lewis a (Le^a^), Lewis b (Le^b^), Lewis x (Le^x^) and Lewis y (Le^y^).^43^.

**Figure 3.**
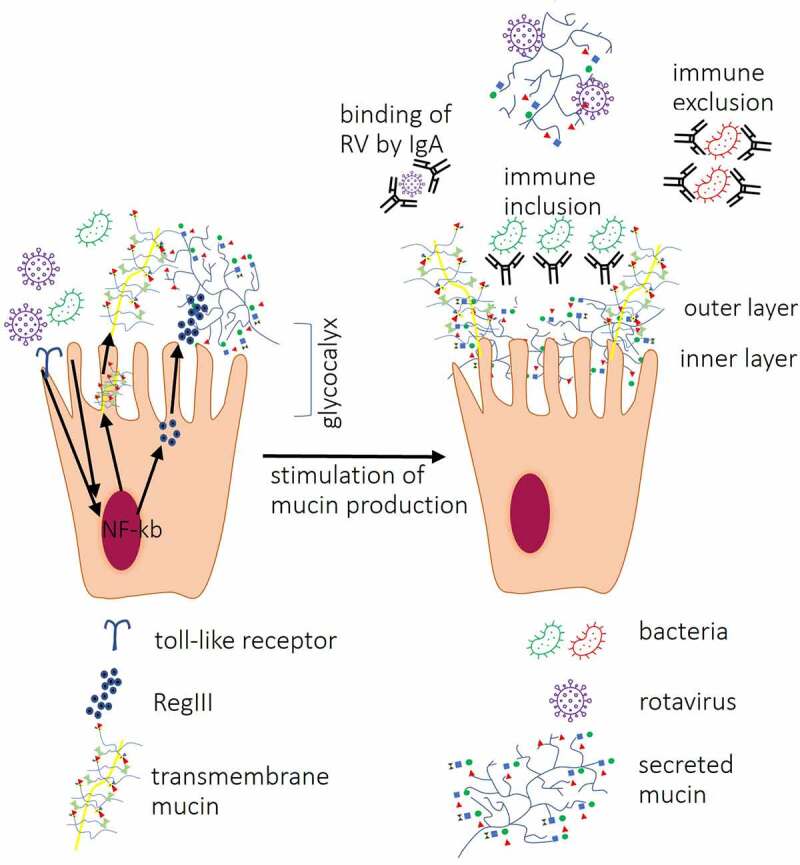
Interactions between IECs and other components of the intestinal mucosa. Interactions between bacterial ligands and Toll-like receptors (TLRs) induce signaling cascades resulting in activation of transcription factor nuclear factor-kappa-chain-enhancer (NF-κb) which lead to increased expression of RegIII proteins, secreted and transmembrane mucins leading to thickening of the mucus layer. Direct contact between RV VP8* and tumor necrosis factor (TNF) receptor associated factor 2 (TRAF2) increases *MUC2* transcription. Produced by goblet cells, secreted mucins form two layers: first, dense firmly attached inner layer directly covering IECs surface, non-penetrable for bacteria. Second, loosely attached layer is a habitat for bacteria. Mucin *O*-glycans within inner and outer layer directly interact with RV and IgA binds RV preventing them to reach IECs. Antibacterial barrier function of mucus layers is further supported by antimicrobial RegIII protein. IgA produced by plasma cells in Peyer’s patches cells block receptors on IEC surface and/or directly bind pathogenic bacteria, immune exclusion, and RV or may facilitate the formation of biofilm (immune inclusion).

The diversity of the GI mucin-type *O*-glycans is determined by the glycan core structure (cores 1–8, with cores 1–4 being most common), type of HBGA precursor and different terminal modifications that can include fucose, galactose (Gal), N-acetylgalactosamine (GalNAc) and SA (Figure 2).^44^ Glycosyltransferases (GTs) provide a critical role in formation of this mucin-type *O*-glycan biodiversity, while inhibition of these enzymes’ activity results in increased mucus permeability.^45^ The expression of secreted ABO(H) antigens and the Lewis b (Le b) antigen is regulated by fucosyltransferase 2 (FUT2), encoded by the *FUT2* gene, while the expression of membrane-associated H antigen is regulated by FUT1.^46,47^ Different sialyltransferases are involved in the addition of terminal SAs to *O*-glycan chains.^48^ Fucosyltransferase 3 (FUT3) is involved in the biosynthesis of Lewis antigens.^49^ Three alleles of *ABO* gene encodes

GTs responsible for converting the H antigen into A and B antigens (alleles A and B), while O allele is an inactive GT that leaves the H antigen unmodified.^50^ Polymorphisms of fucosyltransferase-encoding genes have been found to determine the fucosyltransferase activity.^51^ For example, 14 genotypes of *FUT3* and 10 genotypes of *FUT2* have been recognized only in Chinese population.^52^ Besides, GTs have been found to interact and compete with each other thus affecting the glycoconjugate profile.^53^ In addition, *O*-glycan distribution has been found to vary in different regions of GI tract.^47^ The biodiversity of mucosal *O*-glycans may play an evolutionary important role in protection against pathogens known to interact with certain glycans such as HBGAs resulting in lower susceptibility of mammals.^54^

## O-glycans are important ligands for rotavirus attachment and entry

The main targets for RV infection are IECs located at the tips of intestinal villi.^55^ However, for some strains/genotypes of species A RV (RVA), infection is not limited to IECs, but high-level RVA detection reportedly occurs in extraintestinal tissues including immune cells.^56,57^ Antigenemia and RNAemia have been reported in children with RVA diarrhea,^58,59^ suggesting a mechanism of extraintestinal spread of RVAs to highly vascular organs such as liver, cerebrospinal fluid, spleen and lungs. A study by Azevedo and coauthors demonstrated similar observation in pigs inoculated with human RVA.^60^ However,*^61^ whether RVA infection is associated with efficient viral replication in immune and/or other blood cells or just passive virus uptake and transport remains to be evaluated. Detection of RVA in extraintestinal tissues has been associated with pathology in some studies. For example, along with the expected changes in the intestine following RVA infection of rats, histopathological changes associated with RVA antigens were observed in the liver and lungs.^56^ The same study confirmed the ability of G3P5B[3] RVA to infect porcine alveolar macrophages. Recently, a similar observation has been demonstrated for porcine RVA.^62^ Further, Ciarlet and coauthors observed efficient replication of RVA on cell lines derived from intestine, stomach, breast, bone and lung.^63^ The wide range of cells permissive for RVA replication may be explained by the presence of common attachment sites in all of these cell types, such as integrins that was reported to enable RVA replication in Chinese hamster ovary cells.^63^ However, there are no data on the role of *O*-glycans in extraintestinal replication of RVs.

RV infection requires specific interactions (including virus attachment and entry) between RVs and host cellular attachment sites. RV exposure to the main small intestine proteinase (trypsin) results in the cleavage of the spike protein (VP4) into an N-terminal domain, VP8*, and a C-terminal domain, VP5×.^64^ The VP8* domain binds to host cellular sialylated glycans.^65^ However, proteolytic priming of viral particles is not required for the RV binding but is essential for cell membrane penetration and virus entry into the host cell^66^, suggesting that RV uses different ligands for cell attachment and entry.

There are several surface molecules, such as terminal^3^ and internal^4^ (monosialotetrahexosylganglioside, GM1 ganglioside) sialylated glycans, HBGAs^6^, heat-shock cognate protein (hsc70)^67^, tight junction proteins^68^ and integrins^69^ which have been recognized as ligands for RV attachment and entry into IECs. However, the principal receptor for RV attachment/entry remains to be identified. Several approaches have been implemented to dissect the roles of the aforementioned attachment sites in RV replication. For example, cell treatment with sialidase (neuraminidase, NA) cleaving terminal α2,3-, α2,6-, or α2,8-linked SA residues has been found to significantly decrease attachment of some animal RVA strains (simian SA11 G3P[2], RRV G3P[3], bovine NCDV G6P[1] and porcine OSU G5P[7]) to various cells emphasizing the role of SAs as an attachment site for RVA.^70^ However, most human RVAs (Wa G1P[8], DS-1 G1P[8], ST3 G4P[6] subtype A, K8 G1P[9], S2 G2P[1]) and some animal RVAs (bovine 223 G10P[11], porcine Gottfried G4P[6], equine H2 G3P[12] and FI23 G14P[12]) are not dependent on the presence of sialylated glycans to infect cells.^70^ These initial observations led to classification of RVAs as NA-sensitive and NA-insensitive (NA-resistant) which later was revisited following evidence that NA treatment only removes terminal SA residues but not the internal ones.^71^ A study by Haselhorst and colleagues demonstrated an enhanced replication of “sialidase-insensitive” human RVA Wa G1P[8] after SA (α2,3-, α2,6-, or α2,8-linked) removal.^72^ We have recently demonstrated significantly enhanced replication of porcine RVA RV0084 (G9P[13]) after terminal SA removal with *Arthrobacter ureafaciens* sialidase (cleaves terminal α2,3-, α2,6-, or α2,8-linked SA residues) treatment.^73^ Our lab has also demonstrated that human and porcine RVCs utilize sialylated glycans for binding/attachment.^74^ NA treatment resulted in enhanced porcine RVC Cowden G1P[1] and RVC RV0143 G6P[5] replication, but inhibited the growth of porcine RVC RV0104 G3P[18], further highlighting the role of terminal SA in RV replication.^74^ Thus, our data suggested that terminal SA residues may mask some other attachment sites recognized by RVs including internal sialylated glycans. For example, internal sialylated glycans have been shown to serve as attachment sites for NA-resistant RVs.^4,72,75^ Therefore, recently other strategies have been applied to dissect the roles of sialylated glycans in RV replication. Studies have shown that CRISPR/Cas9 knockout of the solute carrier family 35 member A1 gene (SLC35A1, encoding a key GT essential for SA biosynthesis) led to the loss of sialylated glycans on the cell surface.^76,77^ The latter coincided with loss of NA treatment effects on replication of sialidase sensitive simian RVA.^77^

The interplay between the RV VP8* domain and sialylated glycans is an example of lectin-glycan interactions characterized by low affinity^65,78;^ while lectins with multiple binding sites have a significantly higher affinity to glycans.^79^ The ability of lectins to bind sialylated glycans is of importance since neutralizing Abs developed against the SA-binding domain of VP8* inhibit RVA hemagglutination. Thus, VP8* interactions with various glycans are likely to increase the host cell binding capacity of RV.

There are several lectins of different origin (including invertebrates and plants) that bind *O*-glycans.^80^ Jolly and colleagues demonstrated ability of galactose-specific plant lectins to inhibit RVA replication in a strain-specific manner.^81^ The same study showed amino acid sequence similarity (27%) between RV VP5* and the galactose-binding domain of a plant lectin (*Ricinus* agglutinin) suggestive of similar mechanisms engaged by plant lectins and VP5* while binding host *O*-glycans. Altogether, these data confirm the significance of highly specific interactions between sugar residues on the IEC surface membranes and within the intestinal mucus with carbohydrate-binding proteins of bacteria (discussed in section 4.1), plants and RVs.

The group of HBGAs play a critical role in RV binding/entry into the IECs. HBGAs are a group of glycans and represent a large family of carbohydrates which consists of more than 300 recognized antigens.^82^ First detected on the red blood cell surface, these molecules have subsequently been detected in many tissues including epithelial cells lining the respiratory, gastrointestinal, and reproductive tracts, skin and granular secretions.^83–87^ Along with epithelial cells found to carry high numbers of HBGAs including ABO and Lewis molecules, HBGAs have also been detected in diverse biological fluids such as intestinal content, blood (erythrocytes) and saliva of secretor individuals.^86^ Thus, HBGAs are present in tissues that are in direct contact with the external environment, and the widespread distribution of sialylated glycans and HBGAs may facilitate RV replication outside of the intestine. The role of HBGAs in RV infection has been demonstrated in our recent study whereby inhibition of HBGA synthesis in porcine ileal enteroids significantly reduced replication of human G1P[8] RVA Wa.^73^

The glycan-binding specificity of RVs is usually studied in the context of their interactions with HBGA and SA-containing glycan terminal structures. However, specific recognition of mucin cores has also been demonstrated for RVs of different origin (Table 1).^89,92^

**Table 1.** A summary of glycan cores, SA-containing glycans and HBGA types recognized by different RVA and RVC genotypes in different hosts.

RV species	Host	VP4 genotype	Preferred glycan recognition		
			Mucin core	SA	HBGA
RVA	Human	P[4]	Core 2^88^, does not bind core 4 and 6^89^	SA-independent^3^	Le^b^, H-type 1; A-B- types^6,90,91:^ does not bind A, B, Le^a^, Le^b85^
		P[6]	Does not bind core 2^92^		A, B, H, Le negative, H type 1^92,93^
		P[8]	Core 2, does not bind core 4 and 6^89,92^	Internal SA (GM1)^72,94^	Le^b^, H-type 1^6,89^, Secretor positive. Lewis^95;^ does not recognize A type and H type 1^90,96^ does not bind A, B, Le^a^, Le^b^ and H type 2^89;^ Non-secretor^97,98^
		P[9]	N/A	SA-independent^3^	A type^99^
		P[11]			Type 1 and type 2 precursors^100,101^
		P[14]			A type^89,99^
		P[19]	Core 2, 4 and 6^88,92^		H-type 1^88,92^
		P[25]	N/A	N/A	A type^102^
	Porcine	P[5]	N/A	SA-independent^3^	N/A
		P[6]	Core 2^92^		Does not recognize H-type 1. Recognizes both A and H types ^91,104^
		P[7]	N/A	Terminal SA^3^	H type^104^
		P[10]		N/A	recognizes H-type 1^103^
	Bovine	P[1]	N/A	Terminal SA^63^	N/A
		P[5]		SA-independent^3^	
		P[11]			
	Equine	P[7]		Terminal SA^3^	
		P[12]		SA-independent^3^	
		P[18]			
	Simian	P[1]		Terminal SA^3^	
		P[2]			
		P[3]			
	Murine	P[16]		SA-independent^3,104^	
		P[20]			
	Canine	P[3]		Terminal SA^3^	
	Lapine	P[14]		SA-independent^3^	
	Feline	P[3]		Terminal SA^3^	
		P[9]		SA-independent^3^	
	Turkey	P[17]			
	Ovine	P[15]			
	Chicken	P[17]			
RVC	Porcine	P[1]		SA-independent^74^	A type^74^
		P[5]			H type^74^
		P[18]		Terminal^74^	

N/A – not available.

Clinical and *in vitro* studies have demonstrated that the interactions between RVs and the host *O*-glycans are both RVA/RVC genotype- and HBGA type-specific (Table 1). For example, two RVA genotypes, P[8] (human Wa and RVP) and P[4] (DS1), were shown to recognize both, the Lewis and H-type 1 antigens, while P[6] (ST3) interacted with the H-type 1 antigen only.^105^ The P[9], P[14] and P[25] genotypes bind to the type A antigens, whereas P[11] interacted with single and repeated N-acetyllactosamine – a precursor of human HBGAs.^99,106^ However, some studies have demonstrated contradictory results. For example, several epidemiological studies revealed that children with A-type were predominantly infected with a P[8] RVA.^105,107,108^ This was further corroborated by our results showing that the human RVA Wa strain (G1P[8]) infected and replicated to higher titers in porcine small intestinal enteroids expressing A-antigen.^73^ However, previously, a crystallography assay failed to confirm that human P[8] RVA Wa binds this antigen.^90^ Another study by Huang and coauthors in children did not demonstrate any direct binding of type A antigen, however, it generated strong evidence of association.^6^ The role of HBGA-type A in RV replication has recently been demonstrated in our study whereby porcine RVC Cowden G1P[1] had a higher level of replication in porcine small intestinal enteroids expressing A-antigen while two other genotypes, RV0104 G3[P18] and RV0143 G6[P5] had a preference for H-antigen expressing organoids.^74^

RV-HBGA interactions have been assessed by X-ray crystallography of a P[14] VP8* in complex with the type A oligosaccharide.^91^ Based on these findings, human susceptibility to RV infection is determined (at least partially) by their HBGA phenotypes (Table 1).

Further, while two α1,2-fucosyltransferases responsible for α(1.2) fucosylation have been recognized (FUT1 and FUT2), in 20% of human population the *FUT2* gene is inactive, and such individuals are referred to as non-secretor phenotype (Figure 2).^109^ Thus, while in non-secretor individuals *O*-glycans are expressed only on IECs surface (provided by the activity of FUT1), in secretors individuals (where both FUT1 and FUT2 are active) there are two types of *O*-glycans: membrane-associated and secreted. The difference in activity of FUT1 and FUT2 enzymes results in different linkages between Gal and GlcNAc (1,3 and 1,4 for type 1 and type 2 precursors, respectively).^110^ It is of importance to emphasize that expression of HBGA in the small intestine is FUT2-dependent,^47^ suggesting that non-secretor individuals have a limited glycosylation profile. Clinical studies have not reported RVA infection (P[8] and P[4]) in non-secretor individuals; and even suggested that this phenotype was restricted to P[8] and P[4] RV genotype infections.^93,111,112^ These data was further supported by a study demonstrating selective recognition of human P[11] to H type 2 antigen (H antigen expressed in non-secretor individuals, Figure 2) over H type 1 antigen (expressed in secretor individuals).^113^ However, recent studies demonstrated P[8] and P[6] but not P[4] infection in non-secretors.^97,98^ Other studies have shown a strain-specific recognition of precursors type 1 and type 2 for RV P[11].^100,101,113^ Taken together, RV binding depends on the presence of certain glycan cores, HBGA precursors and terminal sugar residues.

Of interest, FUT2 and Lewis polymorphisms were previously thought to be associated with the low efficacy of RVA vaccines in certain African populations, where the predominant RVA strains as well as FUT2 and Lewis genotype prevalence differ from those in Western populations.^93^ In contrast to these findings, our recent data dispute this hypothesis by providing evidence that attenuated RVA strains lose their selective affinity for certain HBGAs, but their interactions with SAs remained similar to that of the virulent counterparts.^104^ The discrepancies described above are likely due to the different models, approaches and assays used in these studies. Thus, experimental data on distinct RV affinity for individual HBGAs are still scarce and/or somewhat inconsistent necessitating additional research.

## Interactions between RVAs and intestinal epithelial cells

### Mucus layer is the first barrier against RV infection

RV needs to reach the host IEC surface to initiate its replication cycle, which involves complex interactions with various components of the mucosal layer. The ability of viral particles to diffuse through mucus depends on particle size and surface charge.^114^ While strongly charged particles are trapped, neutral particles diffuse through the mucus.^114^ Thus, mucus layer acts as a nonspecific defense mechanism of the host against the negatively charged particles, such as outer surface of RVA particles.^115^ Of importance, MUC2 glycans serve as binding sites for the VP8* domain of the RVA spike protein VP4^116^ which may be due to the widespread distribution of SA residues and HBGAs as a part of the peripheral carbohydrate structures of this mucin.

Both, extracellular (often extensive) and intracellular domains of transmembrane mucins (Figures 1 and 3) play a critical role in protection against pathogenic microorganisms by modulating inflammatory pathways via phosphorylation.^40^ Cleavage and shedding of the extracellular domain have been suggested to play a role of decoy receptors for pathogenic bacteria.^117^ In addition, shedding of the extracellular domain of transmembrane mucins is believed to regulate intracellular signal transduction pathways affecting IEC metabolism^40^ including conformational changes of integrins – another ligand for RV attachment/entry. Shedding of the extracellular domain of transmembrane mucins is regulated by tumor necrosis factor alpha (TNF-α).^118^ In turn, the production of TNF-α is induced by RV infection.^119^ Thus, the host response to RV infection increases the cleavage of the highly glycosylated part of membrane associated mucins which leads to increased numbers of decoy epitopes in the mucus layer.

The protective role of purified mucins isolated from human milk against infection caused by several RVAs including SA11 G3P[2], Wa G1P[8], DS-1 G1P[8] and ST3 G4P[6] has been confirmed *in vitro* and *in vivo*, demonstrating the beneficial role of non-immunoglobulin factors of breast milk against enteric pathogens.^120^ The protective role of mucins depends on the origin of the extracted mucins.^121^ Colonic mucins had a stronger inhibitory effect on RVA replication compared to small intestinal mucins.^121^ Besides, the same study demonstrated a RV genotype-specific inhibitory effect of crude mucins which was most likely due to the glycan preferences of RVs (Table 1). An *in vitro* study has shown that transmembrane mucin, MUC1, also inhibited RVA infection, whereby MUC1 decreased RVA infection caused by NA-sensitive simian SA11 G3P[2] but not by human NA-insensitive Wa G1P[8].^122^ The MUC1 is known to be highly sialylated^123^ suggesting that the protective role of MUC1 against NA-sensitive RVA is provided by SA residues. Thus, while sialylated *O*-glycans and HBGAs present on IECs aid in RVA attachment and infection, those secreted in mucus might act as decoy epitopes interfering with the infection. These data emphasize the role of non-secretor status (no HBGA secreted in the mucus layer) in protection against RV infection. However, research to date has not yet determined the role of mucus layer in protection against RV infection in non-secretor individuals.

The key role of mucin sugars in RVA attachment was demonstrated in a study where the carbohydrate removal has been shown to abolish the protective properties of mucins suggesting that the inhibition of RVA infection is *O*-glycan-mediated.^120,121^ These results corroborate the findings of *in vivo* and *in vitro* studies which demonstrated that the protective role of mucins was associated with the presence of sialylated glycans. Treatment of mucins with NA to remove terminal SA residues led to the loss of its ability to neutralize RVA infection caused by animal SA11 G3P[2], rhesus RV and human Wa G1P[8] RVs *in vitro* and *in vivo*.^121^ However, while the majority of studies have demonstrated protective effects of transmembrane mucins against RV *in vitro* (as an extract), it is unknown whether the direct interactions between transmembrane mucins and RV facilitate RV attachment to IECs or lead to cleavage of its extracellular domain (with RV bound) and removal from the small intestine via peristaltic and villi contractions. Taken together, secreted and transmembrane mucins regulate *O*-glycan-mediated interactions between IECs and RV.

### Rotavirus infection affects mucus composition

Boshuizen and coauthors demonstrated that RVA infection affects the number of goblet cells along the GI tract in a region-specific manner.^124^ While no difference in the number of mucin-producing cells has been found in the ileum of RVA infected mice compared to non-infected controls, in the duodenum and jejunum, the numbers of goblet cells were significantly decreased in the RVA infected animals.^124^ Recently, a study by Engevik and coauthors revealed that RVA depleted the mucin storage in the small but not large intestine and this effect was not due to decreased numbers of goblet cells, further confirming the possibility of direct interactions between RVAs and mucins.^8^ The same study indicated that mucin depletion was detected within first 48 hours after RVA infection suggesting that the preexisting abundance of mucins in the small intestine and the stimulatory effects of RVA infection on mucin expression do not compensate for high levels of mucins needed at the beginning of infection.^8^ Thus, these data suggest that mucin-stimulating factors, such as probiotics, may provide an appropriate tool for disease mitigation in the early stages of RVA infection.

More specifically, RV interacts with all types of mucin cores present in the intestine including core 2, 4 and 6.^116^ For example, increased *MUC2* transcription has been shown to be induced via interactions between RV VP8* and cellular TNF-α Receptor Associated Factors (TRAFs) through TRAF2-NF-kB kinase signaling, emphasizing also the role of NF-kB in the pathogenesis of RV infections (Figure 3).^125^ Increased production of MUC2 has also been found to be one of defense mechanisms against RVA infection in germ-free (GF) mice.^8^

The glycosylation profiles of the intestinal mucins vary in the course of RVA infection of mice, whereby at the beginning [1 day post-infection (dpi)] sulfated mucins were predominant, while by the 4 dpi sialomucins became more abundant.^124^ The ability of extracted mucins to neutralize RVA infection *in vitro* decreased gradually during the course of infection in mice, further confirming the protective role of early mucins against RVA infection.^124^ The same study has shown that at 4 dpi the RVA neutralizing activity of mucins extracted from infected animals was higher compared with their noninfected counterparts, suggesting the stimulating role of RVA in mucin glycosylation. However, it is unknown whether the fucosylation pattern in mice is similar to other animals and humans

Overall, these findings suggest that RVA-host interactions induce the production of mucin *O*-glycans and enhance mucin glycosylation, thereby boosting the protective role of these decoy attachment sites. These studies summarized above confirmed the key role of the mucin concentration/glycosylation at the beginning of the RVA infection. RVA interactions with IECs reduce the amount of mucin decoy attachment sites emphasizing the potential beneficial effects of bacterial mucin-stimulating factors. Besides these factors, mucin concentration and glycosylation status have been found to depend on the diet. High-fat diets were shown to downregulate goblet cell differentiation, glycocalyx formation, and abundance of mucin-stimulating bacteria, while concentration of mucin-degrading bacteria was increased.^126,127^ However, it is unknown whether a high-fat diet is a confounding factor for RV infection.

## Interactions between intestinal epithelial cells and the gut microbiota in the context of RV infection

### O-glycan specific host-gut microbiota interactions shape the gut microbiota composition

In nonpathogenic conditions, there is no direct interactions between members of the gut microbiota and IECs. While both mucus layers of the gut mucosa are penetrable to macromolecules and viral particles, the inner mucus layer remains completely impermeable to bacterium-sized particles.^28^ In addition, there are other components of the innate immune system contributing to the localization and composition of the gut microbiota. For example, host lectins (galectins, C-type lectins and siglecs^128^ are known to regulate the gut microbiota composition via *O*-glycan specific interactions. The inner mucus layer in the small intestine is completely penetrable by bacteria in RegIIIγ (C-type lectin)-deficient mice (Figure 3), whereas in the colon the inner layer completely prevents the contact between bacteria and IECs.^129^ Similarly, bacterial attachment to components of the intestinal mucus is also *O*-glycan specific. Bacterial lectins (mostly present on pili, fimbriae and flagella) have been found to directly bind mucin terminal sugar residues.^25,130^ Selective carbohydrate specificity of bacterial lectins has been demonstrated for several members of the gut microbiota.^11,25,131,132^ Along with beneficial effect of commensal bacteria lectins on the intestinal homeostasis,^133^ their *O*-glycan-specific interactions have also been found to affect RV infection. Selective carbohydrate specificity of plant and bacterial lectins has been shown to block RVA infection *in vitro*.^16,81^ The protective effect of bacterial lectins against RV relies on direct interaction between bacterial lectins and membrane-associated host glycans, thus blocking these glycans from directly binding to RV. However, *in vivo*, the nearly sterile conditions of the inner mucus layer^28^ restrict contacts between bacterial lectins and membrane-associated *O*-glycans, thus limiting the beneficial effect of bacterial lectins against RV infection. In addition, bacterial lectins may exacerbate RV infection by blocking the decoy attachment sites (secreted *O*-glycans) within the outer mucus layer.

Another mechanism that keeps bacteria away from IECs is production of s (secretory) IgA Abs. sIgA Abs bind a broad range of phylogenetically diverse bacteria by recognizing common epitopes such as glycan structure, which further emphasize the role of glycans in host-microbiota interactions.^134^ sIgA possesses several protective mechanisms, including binding to IECs, Ab independent blocking of pathogens and neutralization of bacterial virulence factors (Figure 3).^135,136^ On the other hand, sIgA Abs have been found to promote growth of commensal bacteria, such as *B. fragilis, in vitro* and *in vivo* by facilitating the biofilm formation, thus, providing immune inclusion (Figure 3).^137,138^ *B. fragilis* was also shown to upregulate the expression of *FUT2* gene.^139^ Thus, the known association between the increased abundance of *B. fragilis* in RV-infected individuals^140^ is a protective mechanism against RV infection. Overall, in the absence of pathological process (steady state), there is no direct bacteria-IECs contact, therefore, the term bacterial colonization should be interpreted as a persistent presence of bacteria within the outer mucus layer. However, the overall effect of the gut microbiota on IECs is mediated by multiple mechanisms that do not require direct contact between the gut bacteria and IECs. First, the metabolism of IECs is regulated by interactions between bacteria and transmembrane mucins.^40,117^ Second, the gut microbiota promotes the IEC homeostasis through production of microbial soluble factors (discussed below).^141^

### Microbiota regulates mucus production and composition

Studies have shown that mucus composition of GF animals differs from that of conventional counterparts, whereby the inner mucus layer of GF mouse colon was found to be penetrable to bacteria-sized beads.^142^ However, conventionalizing of GF mice resulted in the impenetrable status of the inner mucus layer.^142^ Gut microbiota has been found to regulate 10% of the host transcriptome including genes encoding cell proliferation and metabolism.^143^ The ability of the gut microbiota to influence glycosylation patterns within the intestine has been studied extensively. The presence of microbiota in conventional mice has been shown to increase glycosylation of secreted mucin MUC2 by upregulation of genes encoding GTs compared to GF mice.^13^ More specifically, *MUC2* transcription is induced by the interaction between the gut microbiota and IECs through NF-κB signaling induction (Figure 3).^144^ Thus, the presence of microbiota in the gut may be considered as a factor increasing protection against RVA infection.^8,145^

Interestingly, the effect of the gut microbiota on mucus composition is enzyme and gut region-specific. For example, presence of microbiota was shown to increase expression of St3gal4 sialyltransferase in the small intestine while decreasing the expression of St3gal6 α2,3-sialyltransferase.^13^ This study also demonstrated that colonization of GF mice led to increased expression of FUT2 in large but not in small intestine^13^, suggesting that these effects are enzyme- and site-specific.

Gut colonization by microbiota has been found to affect relative ratios between different types of *O*-glycans. For example, a significant change in sialylated/fucosylated glycan ratio occurs within the gut in the process of colonization. At birth, the expression of sialylated glycans in ‘aseptic’ gut is relatively high compared to the concentration of fucosylated glycans.^146^ However, the intestinal mucosa of adults is highly fucosylated and characterized by lower expression of SA-containing glycans.^146^ Similarly, Meng and coauthors have shown that the expression of fucosylated epitopes has been gradually increasing after colonization or during recovery from antibiotic treatment.^147^ Taken together, these data may provide an additional explanation for the increased susceptibility of younger individuals to RVA infection compared to adults.

**Figure 4.**
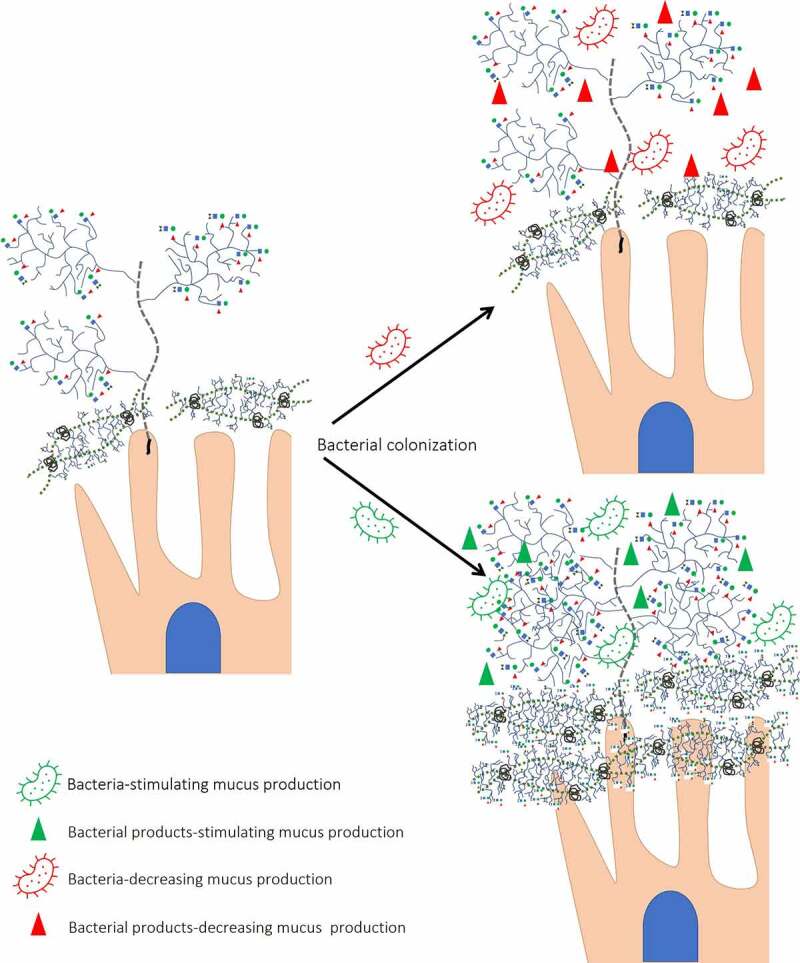
Different effects of bacteria on mucin expression/glycosylation. Presence of glycosyl hydrolases allows bacteria to degrade *O*-glycans decreasing its protective role against RV infection. In addition, bacterial proteases cleave transmembrane mucins. In contrast, bacteria-stimulating bacteria by interaction with transmembrane proteins increases mucin concentration and glycosylation.

The effect of bacterial colonization is also supported by the activity of members of mitogen-activated protein kinases (MAPK) (extracellular signal-regulated kinases and the c-Jun N-terminal kinase).^147^ MAPK activation was found to be dependent on the transmembrane mucin cleavage facilitated by bacterial proteases.^148^ Therefore, while bacterial glycosidases are involved in *O*-glycans consumption (as discussed below), bacterial proteases are involved in IEC metabolism regulated by interaction with transmembrane mucins (Figure 4).

Probiotic bacteria have been found to stimulate mucin production *in vitro* and *in vivo*, where supplementation of rats with a probiotic mixture (*Lactobacilli, Bifidobacteria, and Streptococci)* for 7 days led to a 60% increase of basal luminal mucin content.^149^ This increase coincided with the increased production of gel-forming mucin MUC2, while the expression of genes encoding the membrane-associated MUC1 was only slightly affected. These findings suggest that the probiotics supplementation enhances the production of decoy epitopes which may be helpful in RV binding and elimination. This study also revealed that not only bacterial cells, but also the conditioned media of the probiotic mixture stimulate mucin secretion *in vitro*. Later, this finding was confirmed *in vitro* using *Bacteroides thetaiotaomicron* (*B. thetaiotaomicron*)-derived conditioned media.^150^ Of note, incubation of HT-29 and Caco-2 cells with short chain fatty acids (SCFAs) produced by gut microbiota resulted in increased fucosylation (leading to increased amount of HBGAs).^151^ This emphasized the critical role of the gut microbiota in shaping the intestinal mucus composition. These data suggest that even bacterial metabolites provide beneficial effects on mucin expression further justifying postbiotic use.

A study io mice revealed that while mucin fucosylation protected the host against the invasion by pathogenic bacteria via inhibiting the expression of bacterial virulence genes, increased SA catabolism led to microbial dysbiosis and gut inflammation.^152^ Thus, sialidase producing bacteria may exacerbate RVA infection in two ways: by degradation of SA residues from mucins leading to reduced numbers of decoy epitopes for RVA binding, and by induction of a pro-inflammatory environment. However, since RVA and RVC have been shown to differently interact with terminal SA-residues (discussed earlier), sialidase-active bacteria are likely to have different effects on replication of different RVs.

A study in mice demonstrated that colonization of the intestine not only led to increased mucin production, but also to increased production of longer *O*-glycans (i.e. more sialylated, fucosylated), thus protecting core mucins from bacterial proteases.^13^ Surprisingly, same study reported that some commensal bacteria have an antagonistic effect on mucin secretion.^13^ While colonization of rats with *B. thetaiotaomicron* resulted in increased goblet cell proliferation and higher proportion of *O*-glycans carrying NeuAc or NeuGc residues compared to GF rats, inoculation with another commensal, *Faecalibacterium prausnitzii* attenuated this effect.^153^ Caballero-Franco and colleagues demonstrated that bacterial effect on mucus secretion is genus-specific: while conditioned medium of various *Lactobacillus* species (*L. plantarum, L. acidophilus, L. casei, L. debrueckii*) increased mucus secretion, less appreciable effect was observed for those obtained from single *Bifidobacterium longum* culture and combined *B. longum* + *S. salivarius* cultures.^149^

Thus, while the overall effect of bacterial colonization has been found to upregulate mucin glycosylation, the individual features of bacteria should be taken into account when developing optimal strategies to stimulate mucin glycosylation in order to protect IECs from GI tract infection such as RVA infection.

### Mucus composition shapes the gut microbiome structure

Similar to glycan-binding specificity of RVs (discussed in section 2), the composition of the gut microbiota has also been found to depend on the presence of secreted *O*-glycans.^9,154,155^ Differences in the composition of microbial communities between non-secretors and secretors suggest that more diverse glycosylation profile of secretor individuals might determine gut microbial composition.^154^ Additionally, lower species richness was demonstrated in non-secretor individuals compared to secretors.^155^ Another study demonstrated a higher diversity of the two dominant groups of the human intestinal microbiota: *Eubacterium rectale-Clostridium coccoides* and *Clostridium leptum* in individuals expressing group B/AB compared to individuals expressing group A and H antigens.^9^ Whether this difference is due to the ability of bacteria to preferentially utilize certain HBGAs remains to be evaluated. However, Davenport and colleagues did not find an association between the HBGA/secretor status and gut microbiota composition among 1,500 twins suggesting that further studies are required to dissect the role of the host *O*-glycans in determining gut microbiota composition.^156^

At the family level, the relative abundance of *Prevotellaceae* and *Paraprevotellaceae* was shown to be higher in non-secretor individuals.^157^ Bacteria belonging to *Prevotellaceae* family was found to be associated with increased RV shedding and RV-IgA response in individuals vaccinated against RV infection,^158^ suggesting that this taxa may enhance RV replication. Wacklin and coauthors demonstrated that secretor individuals had a higher richness and diversity of *Bifidobacteria* (including *B. adolescentis*) compared to non-secretor counterparts.^159^ These bacteria has been demonstrated to block RV infection *in vitro* in a genotype-specific manner,^16,160^ suggesting that evaluation of anti-RV effect of bacteria requires the use of different RV genotypes/strains/species.^9^

Mucin sulfation is hypothesized to affect the gut microbiota composition. Of importance, *O*-linked sulfate may be attached to the 6-hydroxyl of N-acetyl-D-glucosamine (6S-GlcNAc) and terminal D-galactose (Gal) carbohydrates at hydroxyl positions 3-, 4- or 6- (3S-, 4S- and 6S-Gal, respectively).^161^ Thus, sulfation is likely to affect antigenicity of carbohydrates for bacteria by masking mucin molecules from bacterial glycosidases preventing their degradation^162^ (discussed in section 4.4). Moreover, RV infection is associated with reduced number of sulfated mucin-containing cells,^124^ suggesting that sulfation of terminal carbohydrates may increase RV binding.

### Mucus as an energy source for microbiota

Taking into account the role of mucin *O*-glycans serving as decoy receptors for RV binding, the ability of the gut microbiota members to degrade these molecules becomes especially important factor for RV infection. Indeed, *O*-glycans, as carbohydrates, may serve as a carbon and energy source for the gut microbial community. An earlier study showed that the absence of the gut microbiota in cecum leads to enlarged cecum full of undegraded mucus.^163^ The ability to degrade mucin molecules has been demonstrated for individual members of all major bacterial phyla of the gut microbiota: *Actinobacteria, Bacteroidetes, Firmicutes* and *Verrucomicrobia* ^164^ as well as for several anaerobic and aerotolerant pathogenic bacteria.^165,166^ Mucin-degrading activity is provided by a group of bacterial enzymes called mucinases which consists of proteases (their role described above), sialidases, glycosidases (glycoside hydrolases: galactosidase, α1–2-fucosidase, α1–3/4-fucosidase) and hexaminidase responsible for the *N*-acetyl-D-glucosamine, galactose and fucose degradation.^167^ Wide distribution of Gram-negative *Bacteroidetes* is associated with its sophisticated enzymatic machinery and the ability to degrade a wide spectrum of complex glycans; while Gram-positive *Firmicutes* possess a highly selective glycan-degrading activity.^168^

Several members of the gut microbiota have demonstrated the ability to utilize sialylated glycans within the mucus layer (Figure 4).^169,170^ Sialidases have been shown to initiate the sequential degradation of mucins *in vitro*. These enzymes are encoded by clustered genes called Nan clusters which have been found in *E. coli* and in members of *Clostridia*, *Bacteroides*, and *Bifidobacterium*. Lack of degradation of SA-containing glycans by *Lactobacillus* members was consistent with the absence or reduced number of copies of the genes encoding for glycosyl hydrolase enzymes.^171^ The ability of mucin-degrading bacteria to decrease protective effects of mucus has been demonstrated *in vitro*, where addition of intestinal murine mucin to MA104 cell line was shown to decrease RVA replication, while pre-treatment of mucin with *B. thetaiotaomicron* or *A. muciniphila* led to increased RVA infection.^8^ In contrast, pre-treatment of mucin with *L. acidophilus* did not affect the release of mucin oligosaccharides.^8^ Thus, degradation of sialylated glycans by some bacteria decreases the role of mucus as a decoy epitope for RV. In addition, terminal SAs mask *O*-glycans from their further degradation by other bacterial enzymes,^172^ suggesting the different role of sialidase-possessing bacteria for SA-sensitive and insensitive RVs.

Bacterial sialidases are divided into three classes: hydrolytic (cleave α2–3-, α2–6- and α2–8-linked terminal SA residues); *trans*-sialidases (α2–6- and α2–8- specificity) and recently discovered intramolecular trans-(IT)-sialidases (specificity restricted to α2–3- linkage).^173,174^ The hydrolitic sialidases with broad spectrum of terminal SA activity were used in a majority of studies describing the role of sialidase treatment in RV infection^3,74^, but some bacteria possess sialidase restriction specificity to α2–8-linked terminal SA residues.^175^ Moreover, other studies also demonstrated sialidase activity against GM-1 ganglioside, suggesting that bacterial sialidases are able to cleave internal SA residues.^175,176^ Several studies have revealed the significant role of gangliosides in RVA infection,^4,94^ indicating that sialidase activity against gangliosides may contribute to replication of certain RVs.

However, the ability of bacteria to release free SAs is not always associated with SA consumption by the same bacterial species.^177^ Some bacteria lacking sialidase activity have been reported to consume Sas. For example, *Clostridium difficile* has been found to consume free Sas due to the presence of the *nan* operon (gene encoding catabolic pathways for SA), but this ability is not mediated by sialidase activity.^177^ These data indicate that presence of sialidases in certain bacteria does not necessarily lead to decreased number of SAs for RV attachment.

Similar to the role of sialylation, mucin sulfation has also been found to protect terminal carbohydrates of *O*-glycans from bacterial degradation. For example, presence of the wide spectrum of sulfatases has been demonstrated to provide a competitive colonization for *B. thetaiotaomicron* and *A. muciniphila* . ^178,179^ These data indicated the role of bacteria with broad spectrum of glycosyl hydrolases (sialidases, sulfatases) necessary for initial degradation of *O*-glycans in decreased protective effects of intestinal mucins against RVA infection *in vitro*.^8^

Mucin degradation has also been reported to be HBGA-specific. While two members of *Ruminococcus* genus were found to produce HBGA-A and HBGA-H degrading alpha-glycosidase sialidases, two *Bifidobacterium* strains were also shown to consume some components from the porcine gastric mucin but do not utilize HBGA-A.^180^ Other human commensal bacteria including *E. coli*, *Enterococcus faecalis*, and *Bifidobacterium* strains have not been found to degrade HBGA-related constituents from porcine gastric mucin.^180^ Taken together, presence of certain bacteria in mucin glycans might be considered as a factor reducing the number of decoy receptors for RVA infection. However, bacterial fermentation of mucin type *O*-glycans results in production of SCFAs which are beneficial for IECs integrity^181^ and immune function.^182^ Thus, more studies are needed to evaluate the role of mucin-degrading bacteria in replication of different RVs.

### RVA/Bacteria interactions with Integrins/Hsc70

Several integrins including α2β1, α4β1, α4β7, αVβ3 and αXβ2 have been shown to be involved in RVA attachment and entry. Graham and coauthors demonstrated that while RVAs of bovine, mice and monkey origin interact with three integrins, α2β1, αXβ2, and αVβ3, none of porcine RVAs (both SA sensitive and insensitive) was integrin-dependent.^183^ Pathogenic and nonpathogenic bacteria have also been shown to interact with cell host integrins. For example, integrin α4β1 was demonstrated to interact with *H. pylori* adhesin, an outer membrane protein (OMP).^184^ Coburn and Cugini demonstrated that αvβ3-integrin-binding protein OMP P66 secreted by *B. burgdorferi* serves as an adhesin molecule allowing *B. burgdorferi* to colonize host cells.^185^ Probiotic extracts bind β3-integrin and Hsc70 (RVA ligands) on MA104 cell surface membranes, limiting RVA attachment and leading to decreased RVA infection.^69^ Additionally, Hsc70 has been identified as a component of the host cells that is utilized by several pathogens such as *E. coli*, *S. typhimurium* and *L. monocytogenes* for their attachment.^186^

Studies have shown a direct connection between the expression of integrins and intestinal mucins, where expression level of integrins on cell surface membranes regulated production of mucins. For example, β1-integrin subunit overexpression was found to reduce MUC5AC but not MUC5B levels (secreted mucins).^187^ Interestingly, α2β1 and β2 integrin expression was shown to be increased after infection caused by human and animal RVAs.^188^ In turn, mucins were shown to regulate integrin conformation.^189^ Changes in integrin conformation led to an increase in their affinity for extracellular ligands including pathogens.^190^ For example, transmembrane mucin MUC1 (its cytoplasmic tail) was demonstrated to affect the integrin-mediated adhesion of *Yersinia pseudotuberculosis*.^191^ Therefore, mucins may affect RVA binding to integrins via conformational changes in integrins. Presence of integrin-binding proteins on beneficial bacteria and/or their capability to interact with Hsc70 have not yet been identified, hence warrant further research.

### Other host factors affecting microbiota-RV interactions

Besides the intestinal mucus composition, several other factors play significant role in RV-microbiota-host interactions. There is strong evidence of the antimicrobial properties of host bile and to a lesser extent digestive enzymes playing a role in RV-microbiota-host IEC interaction.^192^ However, some of the gut microbiota members possess bile tolerance, thus this ability is strain specific. For example, within isolated group of *Lacticaseibacillus rhamnosus* 11 strains were sensitive, 3 – resistant and 8 – tolerant in terms of growth in presence of bile salts.^193^ This agrees with a study for *Bifidobacterium* demonstrated the contrasting results for *B. infantis* and *B. longum*. ^194^ Bile salt hydrolase activity was shown to play a key role in successful adaptation to growing in the presence of bile salts for members of pathogenic, commensal and probiotic bacteria.^195^ Of note, the effect of bile salts on the gut microbiota has been shown to be concentration dependent. While low levels of bile salts resulted in increased abundance of Gram-negative bacteria, high levels coincided with increased proliferation of Gram-positive bacteria and reduction of the Gram-negative *Bacteroides*. ^196^

Members of *Lactobacillus*, *Enteroccocus*, *Bifidobacterium*, *Bacteroidetes* and *Clostridium* have been found to possess a variety of enzymes allowing them to transform bile salts.^197^ Furthermore, GF mice had increased secretion of cholesterol and bile acids compared to their conventional counterparts,^198^ suggesting that the gut microbiota plays a key role in cholesterol metabolism. Overall, studies have shown that the presence of bile (which is secreted into the duodenum and mainly absorbed in ileum) limits bacterial abundance in the small intestine (with 10^4,5^ CFU/mL in duodenum compared to distal part of ileum where populations reach up to 10^7,8^CFU/mL).^199^

Kim and Chang observed that simian RVA SA11 G3P[2] and human RVA Wa G1P[8] replication was reduced by the bile acid treatment of MA104 cells.^200^ However, an *in vivo* study demonstrated the ability of RVA to infect bile duct cells, suggesting that the other components of bile, such as cholesterol, may have an opposite effect on RVA replication *in vivo*.^201^ Since RVA replication is cholesterol-dependent,^202^ the presence of cholesterol in bile but not in bile acid extracts may explain these conflicting results. While little is known about RVC replication, our recent study demonstrated that depletion of cellular cholesterol inhibited replication of porcine RVC Cowden G1P[1], suggesting a similar role of cellular cholesterol in RVC replication.^74^ These data suggest that along with unique features of the mucus layer in the small intestine reducing bacterial density, the presence of bile further limits the ability of some bacteria to provide their beneficial (mucin-, sIgA- stimulating) effects.

## Interactions between microbiota and rotavirus

### Direct rotavirus-microbiota interactions

Several studies have demonstrated expression of glycans by bacteria.^15,16,203,204^ While in mammals, mucins act as a barrier protecting epithelial cells from pathogen attachment, bacterial glycans serve as a defense factor against the host immune system allowing for molecular mimicry and immune evasion, and as virulence factors facilitating host cell invasion.^205^ Similar to mammals, protein glycosylation in bacteria is catalyzed by GTs.^206^ Despite the fact that bacterial GTs have a low nucleotide similarity to mammalian GTs^207^, the enzymatic properties of bacterial GTs are similar to those of human, bovine, mouse and other species.^208^ More specifically, these similarities have been detected for enzymes responsible for HBGA and SA methabolism.^208^ Eventually, expression of bacterial glycans and their recognition by RVs and human glycan Abs suggests a critical role of bacteria expressing glycans in RV infection.^16^

Segmented filamentous bacteria interact directly with RVA affecting its infectivity and disease severity in mice.^209^ Our lab demonstrated the direct binding of *E. coli* Nissle 1917 but not *L. rhamnosus* GG to Wa (G1P[8]) RVA particles or Wa RVA virus-like particles (VP2/4/6) but not to VP2/6 virus-like particles.^210^ The protective role of *E. coli* Nissle 1917 against RVA infection has been evaluated and compared with that of *L*. *rhamnosus* GG, whereby inoculation of GF piglets with *E. coli* Nissle 1917 decreased diarrhea severity and virus shedding after RVA challenge to a greater extent than *L. rhamnosus* GG inoculation.^210^ Recently, we have shown that *E. coli* Nissle 1917 but not L. *rhamnosus* GG bound RVA and RVC and decreased replication of multiple RVA strains *in vitro*. ^16^ Moreover, members of two genera, *Ruminococcus* and *Oxalobacter* that express glycans (A, B, H and Lewis A) on their surface were shown to bind Wa G1P[8] RVA strain.^211^ Thus, bacterial glycans could provide protection against RVA infection *in vivo*.

Recent studies have demonstrated that proteins in probiotic extracts binding to Hsc70 and β3 integrin inhibited RVA infection of MA104 cells by blocking viral adhesion rather than entry.^69^ Studies have also shown that *Enterococcus cloacae* produces glycans that are capable of binding RVA via interaction with the VP8* domain thereby inhibiting its replication.^157^ The ability of HBGA-expressing bacteria to directly bind noroviruses (NoVs) has been shown to protect NoVs from acute heat stress and facilitate NoV infection *in vitro*.^212^ These studies of RVA and NoV infections emphasize that significant differences occur between *in vivo* and *in vitro* infections. In the course of *in vivo* infection, RVA interacts with *O*-glycans on the IEC surface as well as secreted *O*-glycans within the intestinal mucosa and/or glycans present on the bacterial surface and therefore may be removed from the gut by villous movement.^213^ (Figure 3). However, conventional continuous cell cultures do not recapitulate *in vivo* mucus turnover. While gut colonization with commensal microbiota does not result in direct contact between bacteria and the IEC surface^214^, addition of bacteria to continuous cell lines leads to the direct bacterial adhesion and can even cause cell death.^215^ Thus, *in vitro* assays have significant limitations for studying the tripartite RVA-bacteria-host interactions.

In addition to using sialylated glycans as an energy source, some bacteria, such as strains of *E. coli*, *P. multocida* and *B. pseudocatenulatum* are known to possess N-acylneuraminate cytidylyltransferases (CMP-Neu5Ac synthetases), enzymes responsible for SA methabolism.^216–218^ However, production of sialylated glycans in bacteria is not limited to *de novo* biosynthesis and does not require the presence of all enzymes for SA methabolism.^219^ Bacterial sialyltransferases allow bacteria to use an external 5´-monophosphate (CMP)-activated SA (e.g., CMP-*N*-acetyl-neuraminic acid; CMP-Neu5Ac) to synthetize their own SAs.^220^ Therefore, the sialylated glycans on the bacterial cell surface play a role as anti-recognition molecules, allowing bacteria to remain undetected by the host immune system.^221^ Thus, bacterial consumption of *O*-glycans does not necessarily lead to decreased numbers of RVA attachment sites within the intestinal mucosa. However, the role of sialylated glycan-producing bacteria in the context of RVA infection remains poorly understood.

### Microbiota upregulate immune responses to RVA infection

Key roles of microbiota include postnatal immune system development, regulation and promotion of protective immunity against pathogens.^222,223^ sIgA secretion as a part of extrafollicular and T-cell independent Ab responses has been found to be controlled by commensal bacteria.^224^ The increased transcytosis of IgA through IECs is mediated by the polymeric immunoglobulin receptor (pIgR). Some bacteria upregulate plgR expression by the same MyD88-dependent TLR signaling^225^ as has been demonstrated for antimicrobial C-type lectins.^129^ Cash and coauthors observed that the intestine of GF mice had significantly lower concentrations of secreted sIgA, smaller Peyerˈs patches, and reduced RegIIIγ expression.^226^

Several investigators have evaluated the role of gut microbiota in immune responses to RVA infection.^210,227–229^ The beneficial effect of probiotics against virus infection has been studied extensively for lactic acid bacteria.^227,228^ For example, Laino and colleagues showed that *L. delbrueckii* produces immunomodulatory extracellular polysaccharides (EPSs) that allow for interactions between bacteria and host IECs by interacting with PRRs expressed by nonimmune and immune cells.^227^ Most recently, Kanmani and colleagues showed that an innate immune response triggered by TLR3 activation in porcine IECs was differentially modulated by EPS from *L. delbrueckii*. ^228^ Certain probiotic bacteria were demonstrated to upregulate immune responses to RVA vaccine.^229^ Our lab further demonstrated that colonization of gnotobiotic (Gn) piglets with *E. coli* Nissle 1917 resulted in increased RVA-specific IgA Ab titers in serum and intestinal contents after vaccination and challenge with human RVA compared with L. *rhamnosus* GG colonized counterparts.^230^ Supporting our previous observations,^210^ this study suggested that the beneficial effect of bacteria on the immune system is strain-specific. Thus, bacteria provide sIgA-stimulating and strain-specific effects in the intestine.

Antibiotic treatment before RVA inoculation increased the concentration of IgA-Ab producing cells in the intestine which correlated with delayed and diminished RVA infection in a mouse model.^231^ However, in contrast, enhanced RVA infection has been demonstrated in Gn piglets colonized with commensal microbiota and treated with ciprofloxacin compared to the untreated group.^145^ Others demonstrated that the commensal microbiota supports persistent murine NoV infection, whereas antibiotic treatment resulted in diminished viral shedding and viral loads in intestinal tissues.^232^ These inconsistencies may reflect the differential effects of the antibiotics used and presence of antimicrobial resistance genes in certain bacteria.

### RVA infection affects microbiota composition

RVA infection dramatically alters microbiota composition often decreasing the intestinal microbiota diversity, especially of *Proteobacteria* and increased number of opportunistic pathogens.^233^ Our recent study has shown that RVA infection resulted in increased *Firmicutes* abundance which coincided with reduction in *Proteobacteria*.^234^ While the intestinal microbiota in healthy individuals were mainly represented by *Bacteroidetes*, the dominant phylum of the patients with diarrhea was *Firmicutes*.^235^ These changes were not only evident at the phylum level, but variations in microbial composition at the species level associated with RVA infection of children were also noted.^140^

RVA infection increased the abundance of the mucin-degrading *Bacteroides* in mice^8^, humans^233^ and piglets.^236^ The abundance of a member of the genus *Lactobacillus*, lacking mucin-degrading activity, was decreased during the first 3 days of RV infection in mice.^8^ Remarkably, both, mucin stores and gut microbiota composition were fully restored to the pre-infection levels at day 3 post-infection in a strain-specific manner: RVA infection led to increased abundance of *B. fragilis*, whereas the abundance of *B. vulgatus* and *B. stercoris* was decreased. Thus, RVA infection is complicated by the bacteria-mediated decrease in concentration of protective mucins, further facilitating RV replication. Collectively, these factors may contribute to the increased diarrhea severity and virus shedding during first 2–4 days after infection. However, whether the increased abundance of mucin-degrading bacteria is a mechanism that reduces or increases RVA infection *in vivo* is unknown. These bacteria may have a prominent effect on sIgA production as shown for *B. ovatus* ^237^ or carry glycans as decoy epitopes that bind RVA as was demonstrated by our recent study.^16^ Finally, as was shown for *B. thetaiotamicron*, bacteria may stimulate *O*-glycan expression supplying additional decoy epitopes for diverse RVs.^150^

## Conclusions and future perspectives

Despite significant knowledge accumulated in the last 10–15 years regarding RVA pathogenesis, the mechanisms regulating interactions between RVA and host cellular attachment sites remain poorly understood. The discovery of *O*-glycans such as HBGAs as ligands for RVA attachment/entry has expanded our knowledge about the role of host-related factors that influence RVA infection outcomes. Usually the virus-host interactions at or near the cellular surface membranes are the focus of extensive research. However, the important initial interactions occurring within the intestinal lumen and the mucus layer remain understudied. While many body organ systems, including cardiovascular and nervous, are protected from the external environment by a physical barrier, other body systems such as digestive, respiratory, integumentary, and reproductive, combine both physical barrier and absorptive, transport, and exchange functions to protect themselves from the external environment. These functions are supported by the two mucus layers which are the part of the universal innate immune system of aquatic and terrestrial metazoans. In turn, these functions of mucus are promoted by mucus glycoproteins (mucins, including *O*-glycans) that are present not only within the intestinal mucosa in secreted and membrane bound forms, but also on the IEC surface where they serve as sites for RVA attachment/entry (Figure 3).

The gut microbiota regulates mucin production in a strain-specific manner, whereby certain enzymes produce glycans that aid in evasion of host immunity and production of biofilms. On the other hand, mucin-degrading bacteria decrease concentration of *O-*glycans, reducing their protective effects as decoy attachment sites for various pathogens including RV species. Moreover, these gut microbiota members directly bind host *O*-glycans sequestering them to restrict viral pathogen binding. All these features of the gut microbiota, including the stimulation of the immune response to RVA are strain-specific, meaning that the effects of the microbiota on the RVA-host interactions is reliant on the microbiota composition. Thus, targeted modulation of gut microbiota composition including pro-, pre- and postbiotics is an appropriate and innovative approach for RV infection control. Since RV – *O*-glycan interactions are genotype-specific, a similar strategy may be implemented to modulate RV infection outcome. Collectively, RVA infection outcome is a function of multidirectional and complex RV-microbiota-host *O*-glycans interactions with RV genotype-/bacterial species-dependent characteristics. The findings summarized here suggest that RVA adaptation to the host and genetic diversity are influenced by the host and bacterial glycan variability and the ensuing interactions. This knowledge needs to be further evaluated, expanded and considered for development of effective control measures of RVA and other intestinal pathogens.
